# SARS-CoV-2 mutations in Brazil: from genomics to putative clinical conditions

**DOI:** 10.1038/s41598-021-91585-6

**Published:** 2021-06-07

**Authors:** Luis Fernando Saraiva Macedo Timmers, Julia Vasconcellos Peixoto, Rodrigo Gay Ducati, José Fernando Ruggiero Bachega, Leandro de Mattos Pereira, Rafael Andrade Caceres, Fernanda Majolo, Guilherme Liberato da Silva, Débora Bublitz Anton, Odir Antônio Dellagostin, João Antônio Pegas Henriques, Léder Leal Xavier, Márcia Inês Goettert, Stefan Laufer

**Affiliations:** 1 Graduate Program in Biotechnology, Universidade Do Vale Do Taquari - Univates, Lajeado, RS Brazil; 2 Graduate Program in Medical Sciences, Universidade Do Vale Do Taquari - Univates, Lajeado, RS Brazil; 3 Graduate Program in Cellular and Molecular Biology, Federal University of Rio Grande Do Sul - UFRGS, Porto Alegre, RS Brazil; 4 Department of Pharmacosciences, Federal University of Health Sciences of Porto Alegre - UFCSPA, Porto Alegre, RS Brazil; 5 Laboratory of Molecular Microbial Ecology, Federal University of Rio de Janeiro - UFRJ, Rio de Janeiro, RJ Brazil; 6 Graduate Program in Biosciences, Federal University of Health Sciences of Porto Alegre - UFCSPA, Porto Alegre, RS Brazil; 7 Graduate Program in Health Sciences, Federal University of Health Sciences of Porto Alegre - UFCSPA, Porto Alegre, RS Brazil; 8 Laboratory of Acarology, Universidade Do Vale Do Taquari - Univates, TecnovatesLajeado, RS Brazil; 9 Graduate Program in Biotechnology, Centro de Desenvolvimento Tecnológico, Universidade Federal de Pelotas - UFPel, Pelotas, RS Brazil; 10 Laboratory of Cell and Tissue Biology, Pontifical Catholic University of Rio Grande Do Sul - PUCRS, Porto Alegre, RS Brazil; 11 Department of Pharmaceutical and Medicinal Chemistry, Institute of Pharmacy, University of Tübingen, Tübingen, Germany

**Keywords:** Genome informatics, Genetic association study

## Abstract

Due to the high rate of transmissibility, Brazil became the new COVID-19 outbreak epicenter and, since then, is being monitored to understand how SARS-CoV-2 mutates and spreads. We combined genomic and structural analysis to evaluate genomes isolated from different regions of Brazil and show that the most prevalent mutations were located in the S, N, ORF3a and ORF6 genes, which are involved in different stages of viral life cycle and its interaction with the host cells. Structural analysis brought to light the positions of these mutations on protein structures, contributing towards studies of selective structure-based drug discovery and vaccine development.

## Introduction

Early into 2020, a new coronavirus infectious disease (COVID-19) began to rapidly spread around the globe, pushing the World Health Organization (WHO) to declare this outbreak a public health emergency of international concern^1^. Following the first reported case, in Wuhan (on December 19th, 2019), more than 170 million people became infected and over 3.5 million died due to the severe acute respiratory syndrome coronavirus 2 (SARS-CoV-2)^2^. The etiologic agent of this disease is an enveloped single-stranded, positive-sense RNA virus, a member of the betacoronavirus genus presenting a genome of 29,903 kilobases in length^3–5^. Its genome organization consists of an ORF1ab encoding 16 non-structural proteins (NSPs), six accessories proteins^6,7^, a Surface glycoprotein (S), an Envelope protein (E), a Membrane protein (M) and a Nucleocapsid protein (N)^8^. SARS-CoV-2 has been gaining massive attention from the scientific community worldwide, who is putting a great amount of effort into elucidating the mechanisms behind its transmissibility and deciphering how it can be defeated. Distinct socio-economic impacts have been experienced among nations, mainly due to particular political actions in connection with the outbreak. China, Spain, Italy, USA, India and Brazil have assumed leading roles during the pandemic, as they were major epicenters at some point. To date, more than 16 million people became infected and over 461 thousand died in Brazil due to COVID-19. The lack of vaccines and the difficulty of particular members of the government to understand the severity of this disease is increasing our concern, especially considering the appearance of new variants. Therefore, extra attention should be given on countries at such a level, perhaps by monitoring changes in the SARS-CoV-2 genome often associated with the high rate of transmissibility.

In order to monitor the presence of new mutations in the SARS-CoV-2 genome, we applied genomic and structural analysis to evaluate lineages isolated from different regions of Brazil, and mapped where such mutations are located in the protein structures. This information could help understand the impact of these mutations on the stability of the viral proteins, the efficacy of vaccines and to monitor how different the viruses are (here) when compared to other regions.

## Results and discussion

Genomic and structural analysis have brought to light the diversity of the SARS-CoV-2 genome. Several mutations were (and still are being) described within different regions of the world, and the identification of such mutation sites could potentially help the scientific community to build bridges between molecular and disease manifestations. A spike glycoprotein specific mutation (D614G) was the first described to come across advantage/election under positive selection, being associated with higher ability of the virus to infect cells^9^. However, this was not the only mutation identified, as new ones are continuously described; high rates of transmissibility were reported in countries such as the USA, Italy, South Africa, Spain and India, which could be associated with a higher number of mutations in the SARS-CoV-2 genome. The identification of new mutations has been receiving increasing attention worldwide, considering we should be aware of how these could affect vaccine efficacy. As more data is being gathered on viral behavior, we proceeded to annotate and carry out a structural characterization on the distribution of mutations present in genomes isolated from different regions of Brazil in order to understand if there are novel mutations when compared to other countries. We also analyzed the presence of mutations in the spike glycoprotein, and performed analysis on all Brazilian sequences presented in the GISAID database.

### Spike glycoprotein

**Figure 1:**
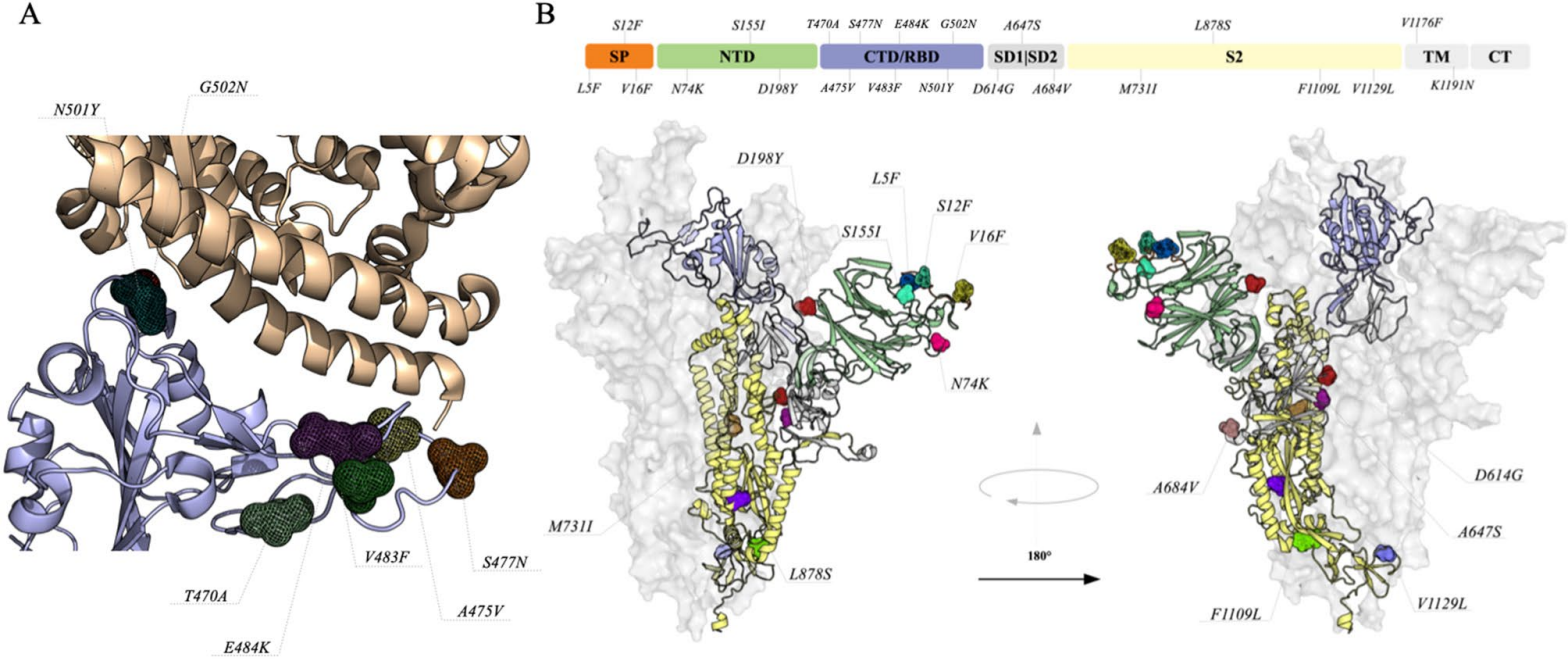
Mutations on the structure of Spike glycoprotein. (**A**) Protein–protein interface of the Receptor binding domains (CTD/RBD) and hACE2. Tertiary structures of proteins are represented as cartoon and the mutation sites are highlighted in different colors and shown as mesh. (**B**) The quaternary structure is shown as surface and cartoon, the mutation sites are highlighted in different colors and shown as mesh. To better show the position of each mutation, we divided the structure in seven domains that are colored as follows; Signal peptide (SP) in orange, N-terminal (NTD) in green, CTD/RBD in purple, Subdomains 1 and 2 (SD1/SD2) in gray, S2 domain in yellow, Transmembrane (TM) and C-terminal (CT) in gray. Mutations at the CTD/RBD are not highlighted in the main structure, since they are presented interacting with hACE2.

The spike glycoprotein is responsible for interacting with the human Angiotensin-converting enzyme 2 (hACE2), driving the process of cell invasion^10–12^. It can be divided into three functional domains: N-terminal domain (NTD), receptor-binding domain (RBD) and S2 domain. Due to its importance in the process of cell recognition and vaccine development, RBD has attracted more attention when it comes to mutations. Our analysis identified the presence of seven mutations in the interface between RBD and hACE2, which are T470A, A475V, S477N, V483F, E484K, N501Y and G502N (Fig. 1A). In addition, combinations of two mutations (S477N+E484K, E484K+N501Y and V483F+E484K) were also observed in sequences from Rio Grande do Sul, Amazonas and São Paulo. Among these, A475V has been associated with decreased sensitivity to neutralizing monoclonal antibodies^13^. Also, S477N, N484K and N501Y were reported to strengthen binding with hACE2^14,15^. Four out of these seven mutations are located at a flexible loop of RBD, and could be important for binding stabilization with hACE2. In addition, mutations in RBD should be monitored due to their possible interferences on the efficacy of recombinant spike protein vaccines. Regarding the other domains, 15 point mutations (L5F, S12F, V16F, N74K, S155I, D198Y, D614G, A647S, A684V, M731I, L878S, F1109L, V1129L, V1176F and K1191N) were also observed (Fig. 1B). Five of these mutations (L5F, S12F, V16F, N74K and S155I) have surface exposure. Although these residues are not in the RBD domain, the proximity could have an impact on cell interaction. Korber and coworkers^9^ recently identified the L5F mutation in 13 countries, and our analysis shows that this particular mutation is also present in Brazil. In addition, mutations on hydrophobic residues in the signal peptide sequence (L5F, S12F, V16F) have been associated with the entry of the spike glycoprotein into the endoplasmic reticulum for viral folding and assembly^16,17^. According to our site-wise evolutionary rate analysis (Supplementary Materials), these positions are more likely to mutate and, among these, D614G was previously described to increase infectivity rates^9^. Five of these positions were predicted as structure stabilizer mutations, V16F, A647S, M731I, L878S and V1129L, presenting ΔΔG values of 2.35, 1.18, 1.18, 4.55 and 1.03, respectively. A647S and V1129L are located at the subunit interface, being potentially involved in the stabilization of the quaternary structure, whereas V16F, M731I and L878S are involved with inter-domain interactions that could present a promotion in tertiary structure stability. Aside from the structural impacts, some mutations require special attention when the goal is to develop vaccines; L5F and K1191N are in sites recognized by Human Leukocyte Antigen (HLA) according to Campbell and coworkers^18^.

### ORF8 protein

**Figure 2:**
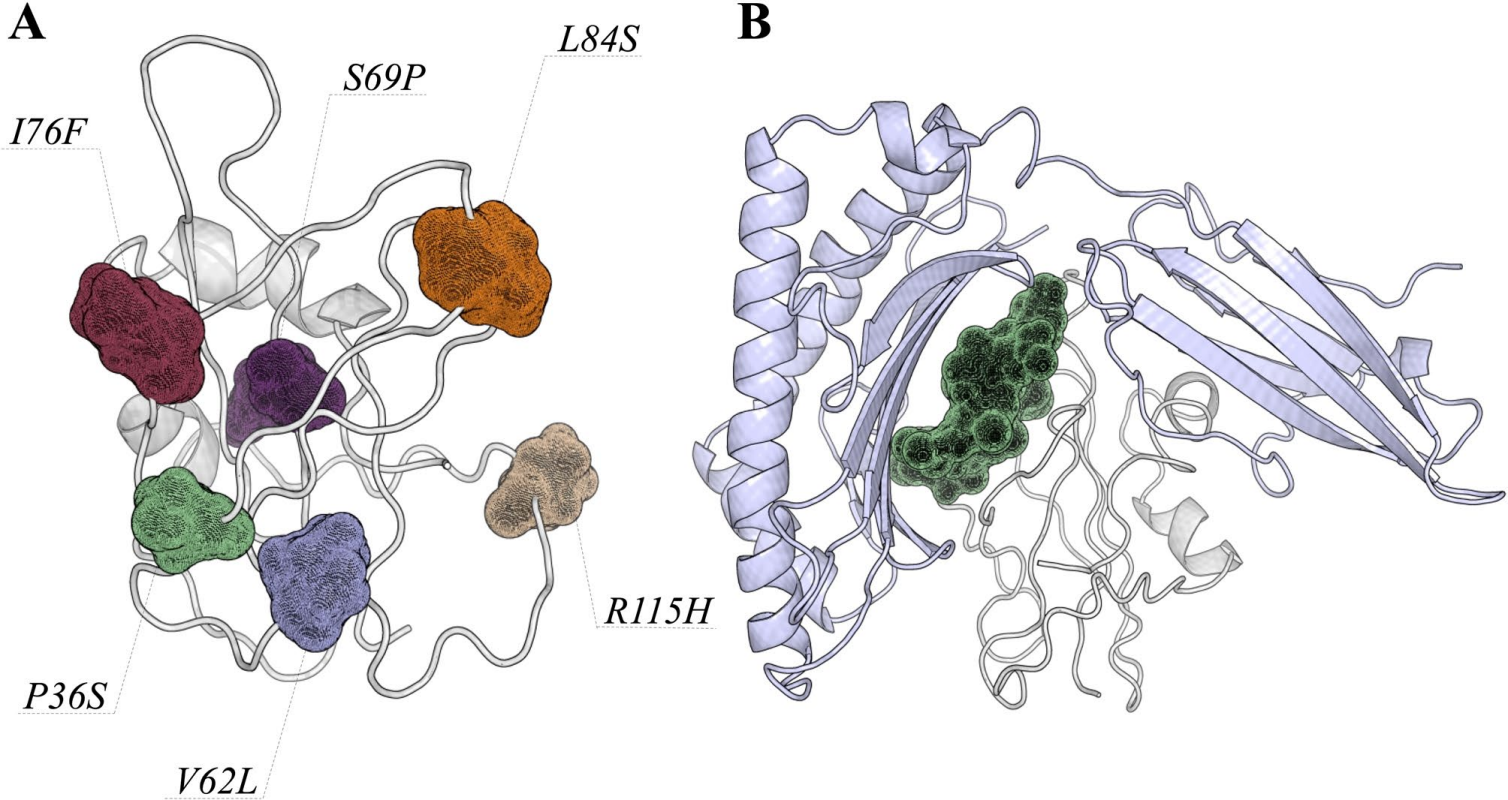
ORF8 protein and its interaction with MHC-I. (**A**) Shows the position of each mutation observed in the SARS-CoV-2 genomes isolated from different regions. The ORF8 structure is shown as cartoon, and the amino acid positions are shown as mesh. (**B**) The most stable conformation of the ORF8:MHC-I complex predicted by the HADDOCK program. MHC-I and ORF8 are shown as cartoon and colored in light blue and gray, respectively. The region from Y73 to V81 is shown as mesh, to highlight where the viral protein is recognized by the host. It is important to observe that mutations in these regions could directly impact on protein–protein interactions.

The ORF8 protein, a member of the betacoronavirus NS8 family, is composed of 121 amino acids. Its function has been associated with viral pathogenicity or replication acting in the host interferon pathway^19–22^. Members of this family present two conserved motifs ranging from 34EDPCP38 and 88INCQ91. Interestingly, SARS-CoV-2 has a mutation on E34D, changing the sequence motif to 34DDPCP38, which may not reflect on a larger impact on protein structure as both amino acids share the same physicochemical properties. On the other hand, we identified six amino acid positions with higher evolutionary rates (P36S, V62L, S69P, I76F, L84S and R115H); V62L was identified in 11 other countries and L84S has been widely observed throughout the world (Fig. 2A). Zhang and coworkers^23^ suggested that the ORF8 protein is able to interfere with the antigen presentation, affecting the process of recognition and elimination of the virus by cytotoxic T cells.

We carried out docking and molecular dynamics simulations to understand how ORF8 protein and MHC-I can interact. Figure 2B presents the residues involved in these protein-protein interactions. Our analysis showed that 11 residues of the ORF8 protein (H28, Y46, Y53, Q72, I74, D75, N78, 79Y, 80T, E106 and H112) are involved in hydrogen bonding to the MHC-I molecule. In order to gain insight and evaluate the stability of the ORF8:MHC-I interaction, we applied molecular dynamics simulations. We specifically analyzed the most representative conformations obtained during the simulation time. Here, cluster 1 presents values of 2.38 nm and 0.28 nm, whereas cluster 2 shows 2.34 and 0.40 of radius of gyration and root mean square deviation, respectively (Supplementary Materials). Analysis of protein flexibility show that the recognition region of the ORF8:MHC-I complex is stable during the simulation time, supporting docking experiments on the binding site (Supplementary Materials). Although we did not observe larger conformational changes between both clusters, it is interesting to highlight that residues ranging from Y73 to V81 are maintaining hydrogen bonds with the MHC-I molecule. This region is close to two mutation sites (I76F and L84S) observed in isolates from different regions of Brazil (and throughout the world). In addition, L84S belongs to the Cytotoxic T Lymphocyte (CTL) linear epitope predicted to be recognized by HLA.

### Envelope protein

The envelope (E) protein is small, composed of 76-109 amino acids, and highly conserved among coronaviruses. It functions as an ion-channeling viroporin, disrupting host membranes and allowing viral release. This protein also interacts with other CoV and host proteins, participating in viral assembly and budding. It is well known that E protein interacts with membrane (M) protein to form the envelope^24–27^. Interestingly, E protein can be detected in abundance inside infected cells during replication, but only a small portion is incorporated into the viral envelope^27^. Inside the host cells, E protein is located mainly in the Golgi, endoplasmic reticulum (ER) and ERGIC (ER-Golgi intermediate compartment)—location of intracellular trafficking between ER and Golgi complex—where it acts on viral assembly and budding, being essential for virion release and, therefore, considered critical for replication^28^.

**Figure 3:**
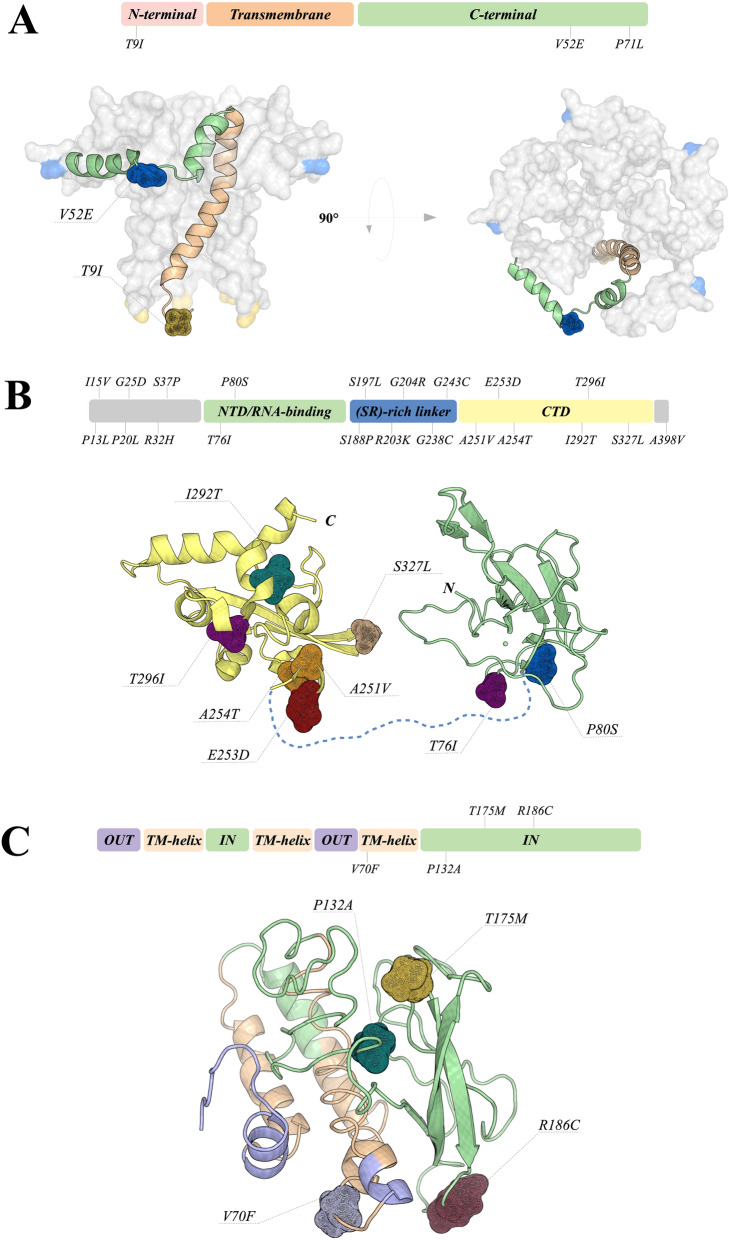
Quaternary structure of the Envelope protein and Tertiary structures of Nucleocapsid and Membrane proteins. (**A**) The modeled structure is shown as surface and cartoon. The regions involved in mutations are highlighted in mesh. The E protein was divided into three domains and colored as follows: N-terminal in light pink, Transmembrane in orange and C-terminal in green. The left figure shows the influence of the mutations in the formation of the viroporin. (**B**) Presentation of crystal structures from the RNA-binding and C-terminal domains of the Nucleocapsid. RNA-binding and C-terminal domains are colored in yellow and green, respectively. All mutation sites are shown as mesh. The linker domain is shown as a dashed line, since it does not have an experimentally determined structure. (**C**) Membrane protein is divided by domains, where most of the mutations are located at the luminal domain. Overall structure is shown as cartoon, and positions involved in mutations are highlighted as mesh.

The E protein is composed of three main domains, the N-terminal (NT), C-terminal (CT) and transmembrane domain (TMD)^27,29^. TMD, located from residues 10 to 35^30^, is responsible for the construction of the ion channel. TMD also presents hydrophobic regions that interact with membrane lipids anchoring the protein into the membrane^25^. The CT domain is a key region for the interaction with M protein, where this association promotes membrane curvature, assuring envelope formation^27^. Furthermore, the CT D-L-L-V motif, also called PDZ-binding motif (PBM), interacts with the PDZ domain of PALS1 (Protein Associated with *Caenorhabditis elegans* Lin-7 protein 1), disrupting tight junctions in epithelial cells by relocation of the protein and, therefore, altering cell polarity and permeability^31^. We have identified three point mutations in the E protein sequence, T9I, V52E and P71L, assigned in Fig. 3A. T9I is located at the negatively charged NT adjacent to TMD (residues 10-35). This mutation indicates a change between threonine (hydrophilic) and isoleucine (hydrophobic). It is characteristic of viroporins to present positively charged residues in the structure, usually lysine and arginine, which assure the anchoring to the membrane by interacting with negatively charged phospholipids^25,32^. Although the NT function is still not clear, NT of viroporins are probably important for ER targeting, together with the TMD hydrophobic region^33^. The substitution of threonine by isoleucine could indicate an enhanced power of interaction with membrane lipids due to its hydrophobic characteristic and its localization; this could also positively modify the capacity of membrane attachment and ER targeting by the E protein. The V52E and P71L mutations are located in the CT domain. V52E mutation is located in a CT position that interacts with membrane lipids^34^ and could alter the interaction with the membrane. P71L is adjacent to the D-L-L-V motif (residues 72-75), a region that binds to the host protein PALS1, facilitating infection. P71L is a mutation that changes a hydrophobic residue into another hydrophobic residue, and has been previously reported in samples collected in the USA^24,26^. It probably promotes none to little effect on protein structure. Although it is possible that the P71L mutation could have some effect on the binding dynamics of the D-L-L-V motif due to its adjacent position, it is less likely to be a deleterious effect, considering how frequently this point mutation is found.

### Nucleocapsid protein

The SARS-CoV-2 nucleocapsid (N) protein is a multifunctional RNA-binding protein involved in essential processes regarding viral RNA transcription and replication. This protein is composed of 419 amino acids and its structure can be divided into three main domains: (i) N- terminal domain (NTD), where the binding of RNA molecules takes place, (ii) a linker domain rich on serine and arginine residues (SR-rich linker), and (iii) a C-terminal domain (CTD) (Fig. 3B), which is also involved in RNA binding modulation. CTD is also required for the process of protein oligomerization, an important step to protect viral genomic RNA. The role of the N protein in the viral life cycle and host infection has been discussed by many research groups, who suggested its involvement in the process of impairment of reproductive and hematopoietic systems via abolishment of pluripotency in human induced pluripotent stem cells^35^, evasion of immune system^36^ and signaling pathway^37,38^. Due to its involvement in different cellular processes, mutations on the N protein should be closely monitored. In addition, nuclear localization signals on the N protein were suggested to be associated with higher fatality rates by coronaviruses^39^, and the accumulation of positive charges could present a contribution to viral pathogenicity; however, no mutations on positive residues were observed in our analysis. Overall, we identified 21 mutation sites, two (T76I and P80S) located at the RNA-binding domain, six (S188P, S197L, R203K, G204R, G238C and G243C) located at the (SR)-rich linker, six (A251V, E253D, A254T, I292T, T296I and S327L) located at CTD, and seven (P13L, I15V, P20L, G25D, R32H, S37P and A398V) located at intrinsic disorder regions. It is interesting to note that the N protein presents the highest number of mutational sites together with ORF3a. Among these, P13L, S188P, S197L, R203K and G204R were widely observed worldwide. The Brazilian sequences P13L, I15V, P20L, G25D, P80S, G238C, G243C and I292T show high evolutionary rates when compared to the other sequence positions. Analysis of the impact of these mutations on protein structure suggests that P80S, A251V and I292T are highly stabilizing, whereas E253D and S327L could be associated with destabilizing mutations. Another important information is that P13L and P80S were predicted to belong to sites of the CTL linear epitopes^18^. Furthermore, the 168-208 region was suggested to be critical for viral assembly and the maturation process to SARS-CoV^40^, so the impact of S188P, S197L, R203K and G204R mutations should be carefully evaluated. Therefore, the high variability of these regions should be taken into account during the process of vaccine development.

### Membrane protein

The membrane (M) protein is composed of 221 amino acids in three transmembrane helices and a cytoplasmic C-terminal domain (Fig. 3C), and is associated with viral assembly. Specific phosphorylation sites in the M protein were suggested to be involved in interactions with the host's cells^38^. According to our results, the M protein presents lower sequence variability, which is in agreement with previous work^41^, suggesting that this protein could be associated with housekeeping functions. The mutations are concentrated in the cytoplasmic C-terminal domain at positions P132A, T175M and R186C, and another one at the third transmembrane helix (V70F).

Site-wise evolutionary analysis suggests that positions P132A and R186C show higher rates of variability, where P132A seems to be a high stabilizer, whereas T175M a destabilizing mutation regarding the tertiary structure. In addition, T175M and R186C mutations are located at the protein surface, which could lead to an impact on protein-protein association since M and N proteins are expected to interact in order to complete viral assembly.

### OFR7a protein

The accessory ORF7a is a type I transmembrane protein composed of 121 amino acids and organized in a transmembrane helix and a luminal domain. Together with nsp1 and nsp3c, ORF7a was suggested to interact with the innate immune system binding directly to BST-2^42^. Due to its possible mechanism, acting as a virulence factor, any mutation should be carefully analyzed.

According to our results, six mutations were observed in sequences isolated from different regions of Brazil, L9P, L10I, E22D, P34S, P84L and V93F (Supplementary Materials). All mutations are located at the luminal domain; however, due to the lack of structural information, we were unable to evaluate the impact on protein structure for L9P, L10I, P84L and V93F. It is important to highlight that L10I and V93F belong to sites of CTL linear epitopes^18^, and should be avoided during the vaccine development process. P34S was described as a high stabilizer mutation, since the presence of a polar side chain could allow hydrogen bond formation to residues H73 and/or Y75.

### ORF6 protein

ORF6 is a small protein composed of 61 residues whose structure is not experimentally known. Among the 27 viral proteins in the coronavirus, the ORF6 protein demonstrated a stronger suppression on primary interferon production and interferon signaling, corroborating previous studies including those on SARS-CoV that characterized the antagonist potential of this protein.

Yuen and coworkers pointed out the ORF6 deletion as an alternative for the production of an attenuated virus-based vaccine^43^. Due to its importance in innate immune suppression, we evaluated the interaction of ORF6 against the transcription factor IRF3 using docking and MD simulations.

Our results suggest that this interaction is stable during the simulation time, and is mainly guided by hydrophobic interactions maintained by residues at the C-terminal of ORF6 (R20, V24, Q56, M58, E59 and D61) (Supplementary Materials). In addition, site-wise evolutionary rate analysis shows that positions E13D, V24F, I32F, I33T and D53Y are more variable (Supplementary Materials).

**Figure 4:**
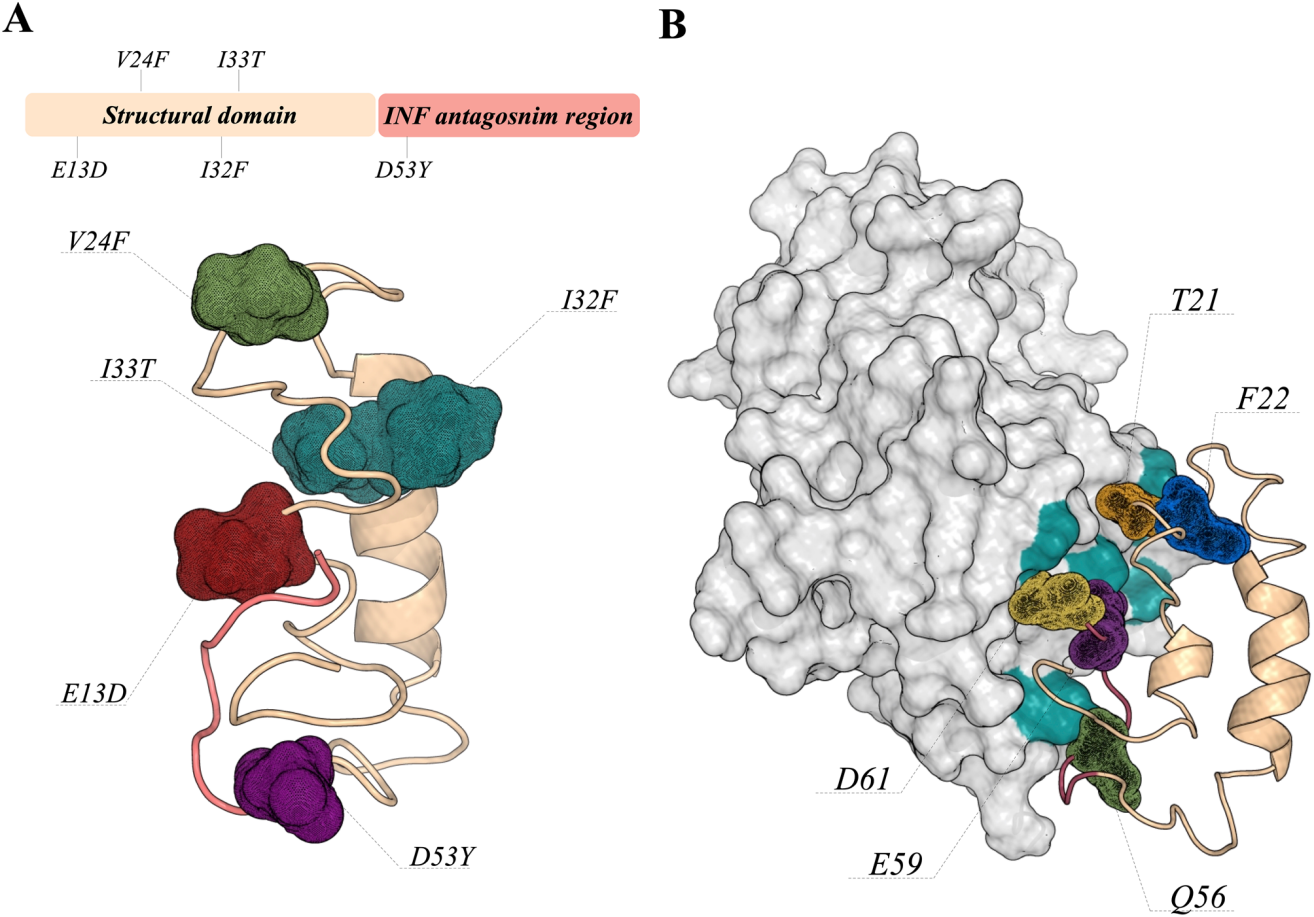
ORF6 tertiary structure and its interaction with the interferon regulatory factor 3. (**A**) Shows the ORF6 structure divided into a structural domain (light orange) and an interferon- binding region (light red). The mutations observed in the genome of SARS-CoV-2 isolated from different regions of Brazil are shown as mesh. (**B**) The predicted binding pose of ORF6:IRF3 complex. IRF3 structure is shown as surface, and the amino acids highlighted in green (Y260, H290, S351, Q354 and R361) are involved in hydrogen bonds with ORF6 residues.

Figure 4 shows the position of these mutations in the model structure of ORF6. Among these positions, it is important to highlight V24F, which could have an impact on the interaction ORF6:IRF3, as this position is located at the interface, and D53Y, which was predicted to belong to CTL linear epitopes^18^.

### ORF3a protein

**Figure 5:**
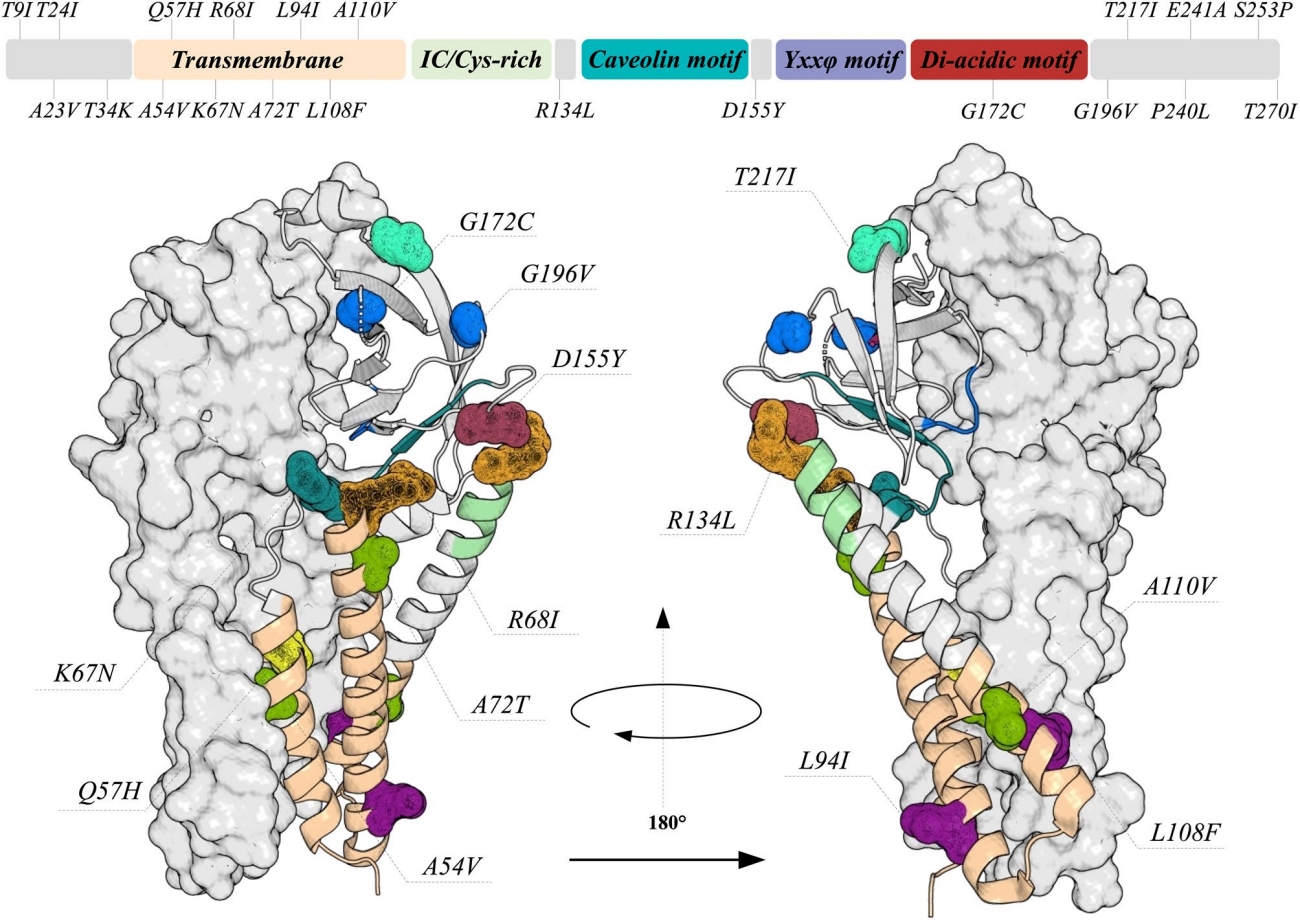
The quaternary structure of the ORF3a protein. The domains of ORF3a are described by the 2D scheme and its colors are highlighted in the structure. The position where mutations were identified are shown as mesh. It is interesting to observe that most of the mutations are located in the transmembrane region. The tertiary structures are presented as cartoon and surface.

The ORF3a protein is an ion transporter composed of 275 amino acids that was associated with different processes during the viral life cycle, such as replication and virulence^44–46^. Its importance in viral pathogenesis was confirmed by Tan and coworkers, who observed that the ORF3a protein up-regulates fibrinogen secretion, which could promote a cytokine storm^47^; however, this may be a result of a cooperative effect based on the interaction among other viral proteins. In addition, this protein was also capable of activating NOD-, LRR- and pyrin domain-containing protein 3 (NLRP3) inflammasome by promoting TNF receptor-associated factor 3 (TRAF3)- dependent ubiquitination of caspase recruitment domain^46^, being an important sensor for the inflammatory stage of COVID-19. Our analysis revealed 21 mutations (T9I, A23V, T24I, T34K, A54V, Q57H, K67N, R68I, A72T, L94I, L108F, A110V, R134L, D155Y, G172C, G196V, T217I, P240L, E241A, S253P and T270I) on the ORF3a protein, where three positions (G172C, G196V and P240L) presented high evolutionary rates (Supplementary Materials). Figure 5 shows these mutations on the structure of the ORF3a protein. These mutations are widely spread along the protein structure, six of them being located at the transmembrane region and another one at the di-acidic motif. It is important to highlight that none of these mutations were observed at the TRAF3- binding motif. However, mutations A23V, T24I and T34K are close to this motif and the impact on ORF3a:TRAF3 interaction should be further investigated. Three mutations (A54V, Q57H and L108F) are at the interface of the subunits and may have effects on protein dimerization. In addition, mutations Q57H, A110V, R134L and P240L are predicted to belong to sites of CTL linear epitopes^18^.

### ORF1a polyprotein

The ORF1a polyprotein is cleaved during the virus life cycle into 11 non-structural proteins, which are the leader protein, NSP2, NSP3, NSP4, 3CL-like protease, NSP6, NSP7, NSP8, NSP9, NSP10 and NSP11. Among these, 3C-like protease is the main target for antiviral development against SARS-CoV-2. Sequence analysis shows that the leader protein, NSP6, NSP9, NSP10 and NSP11 present no mutations when compared to the first genome sequence from Wuhan. However, the other non-structural proteins present at least one mutation. The 3CL-like protease presents two point mutations at position K90R and L205V, and are associated with positive selection. It is important to highlight that no mutation is observed near the active site of the main protease.

NSP7 also shows one mutation at position L71F, which has a high frequency in the Brazilian genomes. NSP8 presents three point mutations (R51C, L95F and A194V), where A194V belongs to sites of CTL linear epitopes^18^; this position was associated with positive selection. Therefore, since the functions of NSP7 and NSP8 were involved in the process of making new copies of viral RNA, any mutation in these proteins should be carefully evaluated. NSP4 presents two mutations, A307Y and H313Y, not predicted to be involved on sites of linear epitopes. Site-wise evolutionary rate analysis shows that NSP2 and NSP3 proteins accounted for most of the mutations in ORF1a, nine being observed in NSP2 and 11 in NSP3 (Supplementary Materials). Among all these mutations, only A194V of the NSP2 was described as being associated with positive selection and to belong to CTL linear epitopes^18^.

### SARS-CoV-2 and the inflammatory process

**Figure 6:**
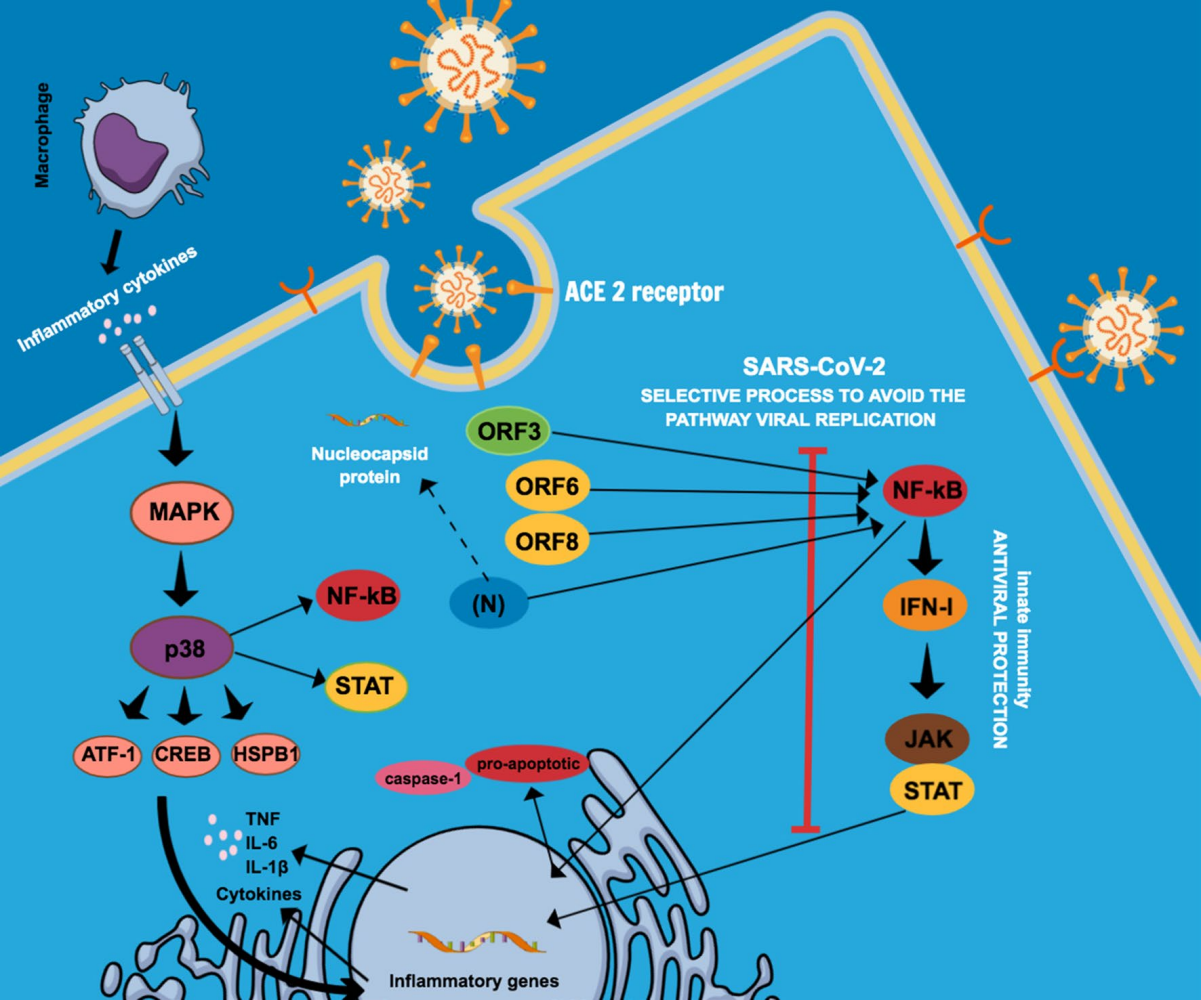
Schematic representation of the SARS-CoV-2 viral infection mechanism by preventing the induction of the IFN-I pathway. The virus inactivates antagonistic members responsive to the viral replication system: SARS-CoV-2 ORF6, ORF8, N and ORF3 inhibit the IFN-I signaling pathway, via NF-κB, being also related to the inflammation activation mechanism. Regarding SARS-CoV, (N) encapsulates the virus genomic RNA protecting and allowing, in the initial stage, the viral replication. ORF3 has been mentioned for its pro-apoptotic activity and is probably associated with a reduction in apoptosis-mediated antiviral defense in infected cells. When the NF-κB activation pathway is inhibited by the virus, another pro-inflammatory response, such as caspase-1, affects the transcription of inflammatory cytokine genes. Mitogen-activated protein kinases (MAPKs) act on the IFN-I pathway and directly on inflammatory elements. From SARS-CoV-2 infection and pro-inflammatory cytokines resulting from the action of macrophages and other antigen-presenting cells (APCs), activation of the p38/MAPK pathway occurs acting on the NF-κB, STAT pathway, thus promoting high expression of pro-inflammatory cytokines, such as IL-6 and TNF-α.

In general, as cells become infected by viral particles, transcription factors (including NF- κB) start to stimulate the expression of IFN-I, a mechanism activated by our innate immune system against viruses. The secreted IFN molecules bind to their specific receptors, initiating the activation of the JAK/STAT pathway and inducing the translocation of transcription factors (responsive to IFN) to the nucleus, establishing an antiviral state^48^. The SARS-CoV-2 infection mechanism, however, uses a selective process that prevents the induction of the IFN-I system, inactivating/blocking antagonistic members responsive to the viral replication system^20^ (Fig. 6).

SARS-CoV-2 ORF6, ORF8, N and ORF3 are potent INF antagonists acting on the early stages of infection. A recent study indicated that their relation to the delayed release of IFNs would hinder the host's antiviral response and benefit viral replication^20^. These proteins are not only capable of inhibiting the IFN-I signaling pathway via NF-κB, but are also related to the inflammation activation mechanism^20^. ORF6 displays a critical antagonistic activity, avoiding the antiviral innate immune response, inhibiting interferon β (IFN-β) production and blocking the expression of STAT1-activated genes to SARS-CoV^20,49^. ORF8 has been considered one of the most relevant proteins related to viral replication in humans, as it is able to inhibit the expression of IFN- β^20,50^. The SARS-CoV-2 N protein acts as an IFN-I antagonist regulating the synthesis and signaling of this route. SARS-CoV has an additional important function that is critical in its antagonism to IFN-I during the viral life cycle: in the initiation stage, the N protein encapsulates the viral genomic RNA, protecting and allowing viral replication^20^ (Fig. 6). Apoptosis, a predominant type of programmed cell death, acts as an important antiviral defense mechanism of the host to control viral infection, being directed to the regulation of inflammatory response. ORF3, which is known for its pro-apoptotic activity, could be potentially associated with a reduction in apoptosis-mediated antiviral defense in infected cells, which could confer an advantage for SARS-CoV-2, as the infection can be relatively mild or even asymptomatic during the early stages, thus allowing the virus to spread more widely^51^. Therefore, this process is in many ways related to the induction of NF-kB, interfering in the IFN-1 route. NF-kB is a transcription factor for the formation of pro- inflammatory cytokines, so when the NF-kB activation pathway is inhibited by the virus, another pro-inflammatory response, such as caspase-1, affects the transcription of inflammatory cytokine genes^44,51^ (Fig. 6).

Another group of proteins which has been associated with SARS-CoV-2 infection and gained attention from researchers is composed of mitogen-activated protein kinases (MAPKs), enzymes that affect the IFN-I pathway and act directly on inflammatory elements^38^. Through SARS-CoV-2 infection and pro-inflammatory cytokines resulting from the action of macrophages and other antigen-presenting cells (APCs), activation of the p38/MAPK pathway acts on NF-kB, STAT pathway and promotes greater expression of pro-inflammatory cytokines such as IL-6 and TNF-α^38,52^. Experimental evidence has shown that, in cured SARS-CoV-2 infected patients, the interferon signal was just mildly activated when compared to severe and moderate infections, and that suppression of the signal pathway related to mRNA translation of the virus occurred. Therefore, down-regulation of the MAPK signaling pathway can be indicative of patient recovery^53^.

In Vero E6 cells (primate kidney cell line), SARS-CoV-2 infection activated the p38/MAPK signaling pathway and the production of various cytokines, and shutdown mitotic kinases, causing cell cycle arrest. Also, there was an upregulation of several components of the p38/MAPK signaling pathway and an increase in phosphorylation of substrates of the p38 pathway in places where regulation occurs 24 hours after infection. Therefore, this may be a reflection of the more advanced stage of viral infection, replication and egress. Among the substrates, we can highlight heat shock protein beta-1 (HSPB1), cAMP response element-binding protein (CREB), transcription factor-1 (ATF-1) and signal transducer and activator of transcription 1-alpha/beta (STAT1)^38^ (Fig. 6). It is still unclear how the inhibition of p38/MAPK could suppress the production of cytokines and impair viral replication during SARS-CoV-2 infection. However, it is possible that the inhibition of p38/MAPK presents multiple mechanisms related to the pathogenesis of COVID-19^38^. Thus, the systematic elimination of IFN modulating functions of the virus becomes an attractive approach for the development of therapies against SARS-CoV-2.

The processes described above corroborate with an alteration in the inflammatory process together with viral replication^54^, triggering the patient's clinical condition. It is important to mention that the elements of inactivation of viral IFN antagonists are an approach for acquiring live attenuated vaccines, and that it has already been studied for influenza A (H1N1) and yellow fever viruses^55,56^.

### SARS-CoV-2 and vaccine development

Several vaccines are being developed against SARS-Cov-2, some currently in clinical trials or already approved for emergency use. There are three main types of SARS-CoV-2 vaccines: inactivated vaccines (Sinovac), RNA vaccines (Pfizer/BioNTech and Moderna) and non-replicating vector vaccines (Oxford/AstraZeneca and Gamaleya Research Institute). The well established technology of inactivated vaccines was used to produce the CoronaVac, designed by Sinovac. This vaccine is composed of inactivated viruses from viral propagation in cells infected with SARS- CoV-2^57^. The clinical trials with CoronaVac demonstrated that it is well tolerated and showed 50.38% efficacy in phase 3, being approved for emergency use in China, Brazil, Indonesia and Chile^57^.

Vaccines produced based on mRNA or adenovirus vector technologies, designed with the gene encoding the SARS-CoV-2 Spike glycoprotein, are presenting higher efficacy than inactivated vaccines, ranging from 62 to 95%^58,59^. However, since the emergence of SARS-CoV-2 variants, with mutations in the RBD of the Spike glycoprotein, uncertainties have taken place regarding the effectiveness of vaccines against these variants. SARS-CoV-2 variants include the B.1.1.7 from the United Kingdom, the B.1.351 from South Africa and P.1 - branch off the B.1.1.28 lineage, from Brazil. The B.1.1.7 variant has eight mutations located in the Spike glycoprotein, including N501Y in RBD domain^60^, while the B.1.351 variant has nine mutations located in the same protein, including E484K and N501Y^61^. The P.1 lineage contains K417T, E484K and N501Y mutations in the RBD of the Spike glycoprotein^62^. Specially for those vaccines designed before the appearance of these variants, mutations in the RBD of the Spike glycoprotein could reflect in loss of vaccine effectiveness. In addition, new mutations are being identified in the RBD such as T470A, A475V, S477N and G502N.

mRNA-based SARS-CoV-2 vaccines exhibit a tolerability and safety profile^63^. There are four types of vaccines developed by Pfizer/BioNTech: BNT162a1, BNT162b1, BNT162b2 and BNT162c2. In two of them, the sequence of Spike glycoprotein is included, while the others contain an optimized RBD. The BNT162b2 is a vaccine of nucleoside-modified mRNA (modRNA) with the full-length of SARS-CoV-2 Spike glycoprotein, encapsulated in lipid nanoparticles. Although this vaccine showed a 95% efficacy (in phase 3) in preventing COVID-19^59^, its effectiveness is not known against new SARS-CoV-2 variants. Analysis of the sera from individuals vaccinated with one dose of BNT162b2 demonstrated that mutations N501Y, A570D and 69/70 deletion combined did not appear to influence the efficacy of the vaccine^64^. Similarly, small effects on neutralization by sera produced by two BNT162b2 doses were observed in SARS-CoV-2 containing the key Spike glycoprotein mutations: N501Y; or 69/70 deletion, N501Y and D614G combination; or E484K, N501Y and D614G combination—indicating that the efficacy of the vaccine may be not be affected by new variants containing these mutations^65^. However, ten of 15 individuals who received the first dose of BNT162b2 vaccine after three weeks had decrease in efficacy of antibodies against the full B.1.1.7 variant (fold change varying between 1.8 and 6.1, with a median of 3.85-fold)^64^. Moreover, an additional decrease in the neutralization of vaccine-elicited antibodies was observed against the B.1.1.7 variant, which presents the mutation E484K^64^.

The vaccine mRNA-1273 developed by Moderna is a LNP-encapsulated mRNA encoding for a full-length Spike glycoprotein. In phase 3 clinical trials, the mRNA-1273 vaccine demonstrated 94.1% efficacy for preventing COVID-19^58^. Studies revealed that this vaccine may also be effective against B.1.1.7 and B.1.351 strains^66,67^, but with a 6-fold decrease in neutralizing levels against the B.1.351 variant from South Africa^66^. Nonetheless, these neutralizing titer levels are still high and the two doses of mRNA-1273 are expected to be protective against the new SARS-CoV-2 strains^66^. Reduction of approximately 2-fold in the neutralization against variant B.1.1.7 was also observed in serum samples from individuals who received the mRNA-1273 vaccine^68^. SARS-CoV- 2 variants carrying the mutations E484K, N501Y or the combination of K417N + E484K + N501Y might be responsible for affecting the effectiveness of the mRNA-1273 vaccine. Individuals who received two doses of mRNA-1273 showed a significant decrease in neutralization activity against variants encoding E484K (1 to 3-fold reduction), N501Y (1.3 to 2.5-fold reduction) and the K417N + E484K + N501Y combination (1.1 to 3-fold reduction)^69^.

Differently from mRNA vaccines, the Covishield (Oxford/AstraZeneca) and Sputnik V (Gamaleya Research Institute) vaccines are based on adenovirus vectors. Covishield (ChAdOx1 nCoV-19) was developed with an engineered chimpanzee adenovirus containing the genetic sequence of the Spike glycoprotein, and showed 82.4% efficacy after two doses^70^. The Covishield vaccine appears to have similar effectiveness against the B.1.1.7 strain of SARS-CoV-2^71^. However, the viral neutralisation by sera induced by the Covishield vaccine might be significantly reduced against the B.1.351 variant from South Africa, when compared to the original virus strain, providing a minimal protection^72^. Due to its lack of efficacy against the B.1.351 variant, Covishield should be carefully evaluated and monitored in Brazil, since mutations T470A, A475V, S477N, V483F, E484K, N501Y, G502N and a combination of two mutations (S477N+E484K, E484K+N501Y and V483F+E484K) have been identified and could also have impact on vaccine efficacy. Moreover, the P.1 variant from Brazil also contains K417T, E484K and N501Y mutations in the Spike glycoprotein^62^. However, no study has been performed to evaluate the effectiveness of the Covishield vaccine in relation to mutation isolates and P.1 variant.

Recently, the company Novavax released preliminary data on clinical trials with its NVX- CoV2373 vaccine, a protein-based vaccine that uses a recombinant SARS-CoV-2 nanoparticle composed of trimeric Spike glycoprotein and a Matrix-M1 adjuvant. This vaccine appeared to be safe and induced immune responses against COVID-19^73^. Preliminary results already include data related to the new SARS-CoV-2 variants. The vaccine is 95.6% effective against the original variant of SARS-CoV-2, while it has 85.6% protection against variants B.1.1.7 and 60% protection against B.1.351^74^.

## Conclusion

According to our analysis, SARS-CoV-2 genomes isolated from different regions of the country present a high number of mutations distributed through several viral proteins. We observed a disproportionality in the origin of these sequences throughout the Brazilian territory. It is worth mentioning that Brazil is a continental size country represented by diverse customs and population profiles. In 537 out of 627 genomes it was possible to check the origin. São Paulo (SP) and Rio de Janeiro (RJ) have the highest concentration of the sequenced genomes, 218 and 120, respectively, while other states present only one sequenced genome (Supplementary Materials). It is possible that the 90 genomes whose origin was not confirmed belong to one of these states, although the information deposited in the database is unclear.

The mechanism by which the virus can develop, replicate and manifest itself with varying degrees of intensity within different Brazilian regions is still an impasse, mainly due to our admixed populations, socio-economic variability and the influence of the environment, such as climatic conditions^75–77^. Due to the high number of cases, Brazil has become an important reference point to monitor the appearance of new mutations and to evaluate the efficacy of vaccines that are under clinical trials. It is important to highlight the high number of mutations in structural proteins such as S and N, as they have been used as targets for vaccine development and for molecular-based detection kits. Among the mutations observed in the SARS-CoV-2 genome, we should be aware of the appearance of (not single, but) double mutations at the interface of hACE2:RBD as observed in individuals from Rio Grande do Sul, Amazonas and São Paulo. These combined mutations could be a new challenge for the efficacy of vaccines. Another important consideration is the lack of public policies and the lack of adherence to social distancing by the Brazilian population, which could increase the probability of the emergence of new variants. Overall, sequence analysis revealed that the mutations observed in different regions of Brazil are positioned at sites of CTL linear epitopes and should be further investigated regarding their possible impacts on the development of new vaccines. In addition, we only described the impact of mutations in the proteins as they were isolated; however, the assembly process of the virion requires protein-protein interactions that are difficult to evaluate based on computational methods. Based on our analysis, we were able to highlight SARS-CoV-2 proteins with the highest evolutionary rates and point out where their mutations are located in the protein structure. This data shows how structural biology, combined with genomics, can be applied to better understand the viral variability, and may be useful in studies of structure-based drug discovery and vaccine development. In addition, clinical practice will be a mirror for these genetic mutations of the virus, where different patterns of clinical manifestations will probably be observed. Important clinical implications must be understood about admixture populations within geographical regions from different parts of the world for the interpretation and design of clinical trials. This heterogeneity influences the practice of clinical genetics, genomic medicine and drug prescriptions when compared to homogeneous populations.

## Materials and methods

### Genome data, open reading frames (ORF) prediction and functional annotation

The complete sequences were obtained from the GISIAD database. The viral’s longest ORF (coding sequences and amino acids) was obtained according to the Markov Model with TransDecoder^78^. The parameters for prediction of Long ORFs and transcripts were modified to keep viral sequences with more than 15 amino acids' length. To refine and identify all ORFs with functional significance, all genomes were annotated according to the sequence of SARS-CoV-2 from Wuhan (NC_045512). The sequences were annotated with OmicsBox using the proteome of SARS-CoV-2 from Wuhan as reference and the tools from EggNOGg mapper, InterProScan and Gene Ontology.

### Measure of amino acid rates

In order to calculate site-specific evolutionary rates for all annotated sequences, we used the Likelihood Estimation of Individual Site Rates (LEISR) method, implemented into the HyPhy program, following the protocol described by Sydykova and coworkers^79^. All sequence alignments and phylogenetic trees were performed using the MAFFT online program with default parameters.

### Crystallographic structures and comparative modeling

In order to identify where the mutations are located in the protein structures, we used the PDB IDs 6VSB (Spike glycoprotein), 6YUN and 6VYO (Nucleocapsid), 6XDC (ORF3a) and 6W37 (ORF7a). We built the models for the sequences that do not have solved structures and for the mutations observed near the active sites and subunit interfaces using the MODELLER 9v23 program. The comparative modeling protocol consisted of the generation of 10 models, where one model for each gene sequence was selected. All models were submitted to the DOPE energy scoring function^80^ implemented in the MODELLER 9v23 program aiming to select the best structures. MOLPROBITY webserver^81^ and PROCHECK^82^ were used to verify and validate the stereo chemical quality of the models.

### Molecular dynamics simulations

The molecular mechanics (MM) and molecular dynamics (MD) simulations of the ORF8:MHC-I and ORF6:IRF3 enzyme complexes were carried out using the Amber99SB force field^83^ implemented in GROMACS 2016.3^84^. Periodic bound conditions were applied, and the number of particles, pressure and temperature were maintained constant (NPT ensemble) during the production phase. Periodic boundary boxes of the ORF8:MHC-I and ORF6:IRF3 systems were composed of 24,538 and 20,857 water molecules, respectively. A V-rescale^85^ thermostat was employed to maintain the system at constant temperature using a coupling time of 0.1 ps, and the Berendsen barostat^86^ was applied to ensure the system pressure was maintained at 1 bar. The LINCS algorithm^87^ was implemented to constrain all the covalent bonds involving hydrogen atoms so that the systems could evolve in a time step of 2 fs. Initial velocities were randomly assigned according to a Maxwell-Boltzmann distribution at 50 K and the temperature was increased up to 298.15 K in four steps (50 K, 150 K, 250 K and 298.15 K). van der Waals interactions were computed with a 12-6 Lennard-Jones potential using a twin range cut-off of 8.0/10.0 Å. Neighbor searching was performed using a Verlet-based cut-off scheme updated every 25 steps with a 10.0 Å cut-off. The macromolecule was fully solvated using the TIP3P model^88^ in a cubic box extending 10.0 Å from the macromolecule surfaces. The systems were submitted to steepest-descent energy minimization up to a tolerance of 1,000 kJ mol^−1^ nm^−1^ to remove close contacts of Van der Waals forces. The equilibration phase lasted for 5 ns followed by the 100 ns of production phase. The root mean square deviation was computed to evaluate the and for the protein-protein complex stability the radius of gyration was calculated. All analyses were carried out using the last 90 ns of each simulation, and were performed with the GROMACS programs and Geo-Measures plugin^89^.

### Protein–protein docking simulations

In order to evaluate the best conformation and binding affinities of the ORF8:MHC-I and ORF6:IRF3 complexes, we applied a flexible protein-protein approach implemented in HADDOCK program^90,91^. The (active) residues involved on protein-protein interactions were predicted according to the CPORT analysis, where the active residues for ORF8 were M1, L7, G8, I10, T11, T12, A15, F16, E19, P30, Y31, V32, V33, D34, D35, P36, C37, P38, I39, H40, F41, Y42, S43, K44, R48, R52, P56, L57, E59, V62, Y73, D75, I76, Y79, T80, S103, F104, Y105, E106, D107, F108, L109, Y111, F120 and I121, while for ORF6 were M1, F2, F7, A12, E13, L15, L16, I17, M19, R20, T21, F22, K23, V24, S25, I26, W27, N28, D30, Y31, I32, N34, L35, I36, I37, L40, I60 and D61. The docking protocol in HADDOCK comprises three main steps, which are (i) rigid body energy minimization, (ii) simulated annealing and (iii) refinement in explicit solvent. The best complexes were selected for further analysis based on HADDOCK score.

## Supplementary Information

Supplementary Information.
