# Supplementary material for: SARS-CoV-2 mutations in Brazil: from genomics to putative clinical conditions
Source: Sci Rep. 2021 Jun 7;11:11998. doi: 10.1038/s41598-021-91585-6 (PMC8184806; doi:10.1038/s41598-021-91585-6)
Supplement: Supplementary file 1 — Supplementary Information. [file 41598_2021_91585_MOESM1_ESM.docx]

**SARS-CoV-2 mutations in Brazil: from genomics to putative clinical conditions**

Luis Fernando Saraiva Macedo Timmers,^1,2^* Julia Vasconcellos Peixoto,^3^ Rodrigo Gay Ducati,^1^ José Fernando Ruggiero Bachega,^4^ Leandro de Mattos Pereira,^5^ Rafael Andrade Caceres,^4,6,7^ Fernanda Majolo,^1^ Guilherme Liberato da Silva,^8^ Débora Bublitz Anton,^1^ Odir Antônio Dellagostin,^9^ João Antônio Pegas Henriques,^1,2^ Léder Leal Xavier,^10^ Márcia Inês Goettert,^1,2^ Stefan Laufer^11^*

**Affiliations**

^1^Graduate Program in Biotechnology, Universidade do Vale do Taquari - Univates, Lajeado, RS, Brazil.

^2^Graduate Program in Medical Sciences, Universidade do Vale do Taquari - Univates, Lajeado, RS, Brazil.

^3^Graduate Program in Cellular and Molecular Biology, Federal University of Rio Grande do Sul - UFRGS, Porto Alegre, RS, Brazil.

^4^Department of Pharmacosciences, Federal University of Health Sciences of Porto Alegre - UFCSPA, Porto Alegre, RS, Brazil.

^5^Laboratory of Molecular Microbial Ecology, Federal University of Rio de Janeiro - UFRJ, Rio de Janeiro, RJ, Brazil.

^6^Graduate Program in Biosciences, Federal University of Health Sciences of Porto Alegre - UFCSPA, Porto Alegre, RS, Brazil.

^7^Graduate Program in Health Sciences, Federal University of Health Sciences of Porto Alegre - UFCSPA, Porto Alegre, RS, Brazil.

^8^Laboratory of Acarology, Tecnovates, Universidade do Vale do Taquari - Univates, Lajeado, RS, Brazil.

^9^Graduate Program in Biotechnology, Centro de Desenvolvimento Tecnológico, Universidade Federal de Pelotas - UFPel, Pelotas, RS, Brazil

^10^Laboratory of Cell and Tissue Biology, Pontifical Catholic University of Rio Grande do Sul - PUCRS, Porto Alegre, RS, Brazil.

^11^Department of Pharmaceutical and Medicinal Chemistry, Institute of Pharmacy, University of Tübingen, Tübingen, Germany.

***Corresponding authors: Luis F. S. M. Timmers (**[**luis.timmers@univates.br**](mailto:luis.timmers@univates.br)**) / Stefan Laufer (**[**stefan.laufer@uni-tuebingen.de**](mailto:stefan.laufer@uni-tuebingen.de)**)**

**Supplementary Materials**

*Molecular dynamics simulations*

To evaluate the conformational changes of ORF8:MHC-I and ORF6:IRF3 complexes, and the stability of the binding pose predicted by molecular docking experiments, we carried out classical molecular dynamics simulations. The distribution of conformations was analyzed based on the radius of gyration and the root mean square deviation was weighed by a probability density function. In addition, the identification of mobile and rigid substructures during the simulation time was predicted using MDLOVOFit program. According to our analysis, 60% of ORF8:MHC-I complex presents less than 2.0 Å of RMSD, and the predicted binding region is highly stable (Fig. S1A). The same pattern could be observed for ORF6:IRF3 complex, where 70% of the structure presents less than 1.0 Å of deviation (Fig. S1B).

**Fig. S1**. **Distribution of the mobile and rigid substructures during the simulation time**. (**A**) and (**C**) show the RMSD as a function of the fraction of the atoms considered in the alignment for ORF8:MHC-I and ORF6:IRF3, respectively. (**B**) and (**D**) illustrate the distribution of structures after alignment for ORF8:MHC-I and ORF6:IRF3, respectively. The more stable regions are colored in blue, whereas the most flexible regions are colored in red. It is important to observe that biding regions predicted by docking experiments remain stable during the simulation time
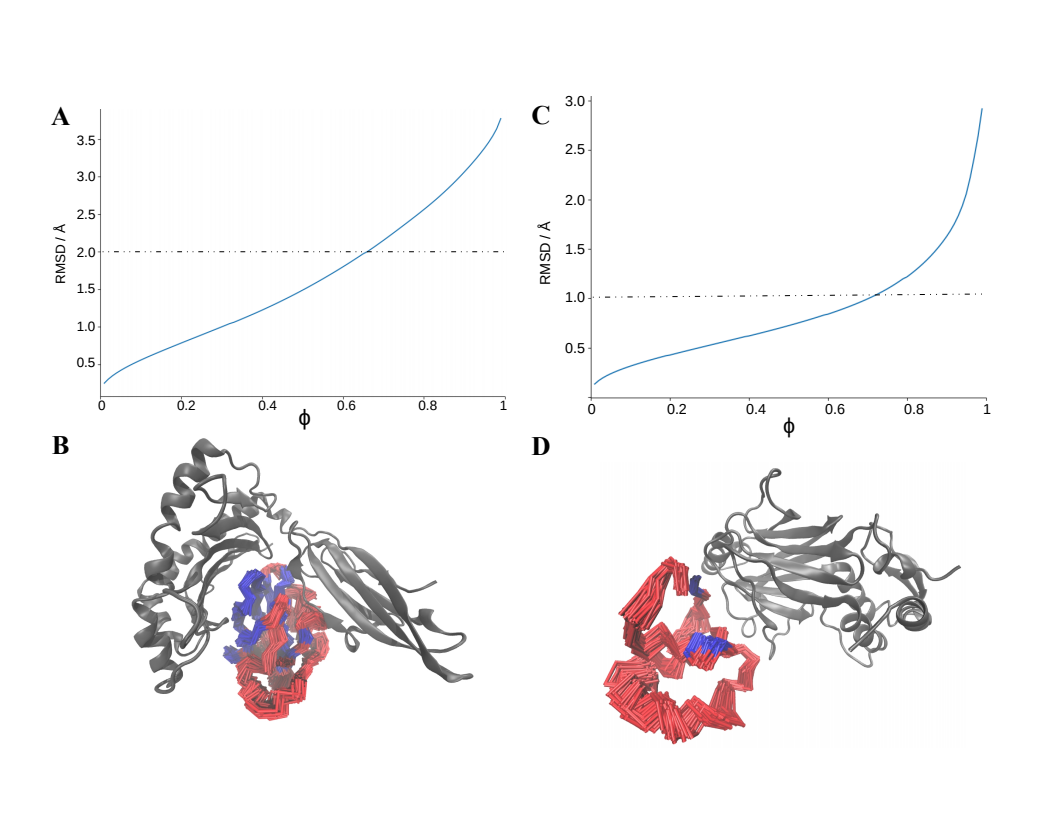


All analyses of protein-protein interactions were performed using PDBePISA, submitting the most conformations of ORF8:MHC-I and ORF6:IRF3 complexes. Fig. S2A and S2B illustrate the conformational ensemble for both systems. During the simulation time, it is possible to observe that ORF8:MHC-I has a more spread plot, presenting two representative conformations, whereas ORF6:IRF3 shows only one stable conformation during the simulation time.


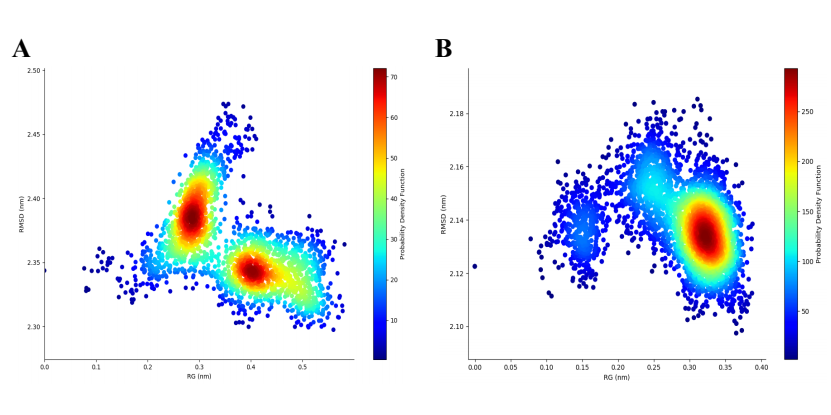


**Fig. S2**. **Description of ORF8:MHC-I and ORF6:IRF3 conformational ensembles**. (**A**) The probability density plots of ORF8:MHC-I, and (**B**) ORF6:IRF3.


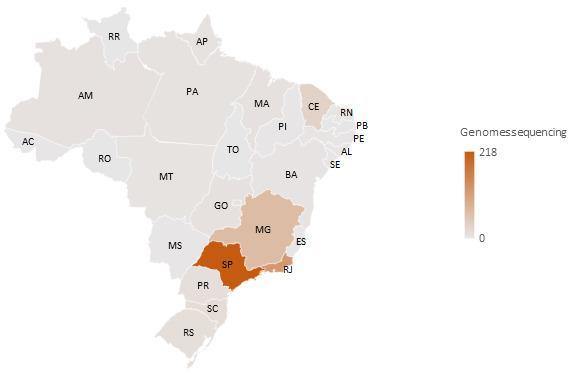


**Fig. S3**. Distribution of sequenced genomes. Brazilians states are colored by the number of sequenced genomes deposited in the GISAID database.

**Table S1**. Estimation of the site-wise evolutionary rates for each SARS-CoV-2 protein.

| **Protein** | **Mutation** | **Rate** |
| --- | --- | --- |
| Spike glycoprotein | L5F | 0.66 |
|  | S12F | 0.35 |
|  | V16F | 0.47 |
|  | N74K | 0.37 |
|  | S155I | 0.37 |
|  | D198Y | 0.54 |
|  | D614G | 0.99 |
|  | A647S | 0.44 |
|  | A684V | 0.44 |
|  | M731I | 0.36 |
|  | L878S | 0.68 |
|  | F1109L | 0.68 |
|  | V1129L | 0.46 |
|  | V1176F | 0.52 |
|  | K1191N | 0.44 |
| ORF8 | P36S | 0.97 |
|  | V62L | 0.48 |
|  | S69P | 0.51 |
|  | I76F | 0.43 |
|  | L84S | 0.68 |
|  | R115H | 0.61 |
| Envelope protein | T9I | 0.38 |
|  | V52E | 0.66 |
|  | P71L | 0.98 |
| Nucleocapsid phosphoprotein | P13L | 0.76 |
|  | I15V | 0.65 |
|  | P20L | 0.79 |
|  | S21T | 0.30 |
|  | G25D | 0.94 |
|  | R32H | 0.42 |
|  | S37P | 0.30 |
|  | T76I | 0.41 |
|  | P80S | 0.74 |
|  | S188P | 0.30 |
|  | S197L | 0.31 |
|  | R203K | 0.36 |
|  | G204R | 0.54 |
|  | G238C | 0.97 |
|  | G243C | 0.97 |
|  | A251V | 0.42 |
|  | E253D | 0.30 |
|  | A254T | 0.43 |
|  | I292T | 0.59 |
|  | T296I | 0.40 |
|  | S327L | 0.31 |
|  | A398V | 0.42 |
| Membrane protein | V70F | 0.35 |
|  | P132A | 0.78 |
|  | T175M | 0.41 |
|  | R186C | 0.67 |
| ORF7a | L9P | 0.39 |
|  | L10I | 0.23 |
|  | E22D | 0.30 |
|  | P34S | 0.98 |
|  | P84L | 0.50 |
|  | V93F | 0.28 |
| ORF6 | E13D | 0.70 |
|  | V24F | 0.52 |
|  | I32F | 0.80 |
|  | I33T | 0.72 |
|  | D53Y | 0.93 |
| ORF3a | T9I | 0.22 |
|  | A23V | 0.21 |
|  | T24I | 0.22 |
|  | T34K | 0.22 |
|  | A54V | 0.21 |
|  | Q57H | 0.21 |
|  | K67N | 0.26 |
|  | R68I | 0.30 |
|  | A72T | 0.21 |
|  | L94I | 0.29 |
|  | L108F | 0.29 |
|  | A110V | 0.21 |
|  | R134L | 0.29 |
|  | D155Y | 0.33 |
|  | G172C | 0.64 |
|  | G196V | 0.64 |
|  | T217I | 0.22 |
|  | P240L | 0.97 |
|  | E241A | 0.23 |
|  | S253P | 0.20 |
|  | T270I | 0.22 |
| ORF1a / NSP2 | A208T | 0.51 |
|  | S318L | 0.41 |
|  | P371S | 0.97 |
|  | R398C | 0.60 |
|  | D589V | 0.60 |
|  | V649F | 0.53 |
|  | A687V | 0.50 |
|  | V774F | 0.53 |
|  | E797K | 0.50 |
| ORF1a / NSP3 | P971S | 0.97 |
|  | H1160Y | 0.55 |
|  | A1204T | 0.51 |
|  | P1207L | 1.00 |
| ORF1a / Papain-like | Y1814C | 0.76 |
|  | T2087I | 0.50 |
|  | H2092Y | 0.55 |
|  | N2162D | 0.42 |
|  | R2471I | 0.63 |
|  | A2554V | 0.50 |
|  | T2648I | 0.50 |
| ORF1a / NSP6 | A3070V | 0.50 |
|  | H3076Y | 0.55 |
| ORF1a / 3C-like protease | K3353R | 0.54 |
| ORF1a / NSP7 | L3930F | 0.73 |
| ORF1a / NSP8 | R3993C | 0.60 |
|  | L4037F | 0.74 |
|  | A4136V | 0.50 |
